# A Study of the Drift Phenomena of Gate-Functionalized Biosensors and Dual-Gate-Functionalized Biosensors in Human Serum

**DOI:** 10.3390/molecules29071459

**Published:** 2024-03-25

**Authors:** Yunjia Song, Nan Chen, Tine Curk, Howard E. Katz

**Affiliations:** Department of Materials Science and Engineering, Johns Hopkins University, 206 Maryland Hall, 3400 North Charles Street, Baltimore, MD 21218, USA; ysong57@jhu.edu (Y.S.); nchen20@jhu.edu (N.C.)

**Keywords:** organic electrochemical transistors, biosensors, protein detection, drift, modeling

## Abstract

In this paper, we study the drift behavior of organic electrochemical transistor (OECT) biosensors in a phosphate-buffered saline (PBS) buffer solution and human serum. Theoretical and experimental methods are illustrated in this paper to understand the origin of the drift phenomenon and the mechanism of ion diffusion in the sensing layer. The drift phenomenon is explained using a first-order kinetic model of ion adsorption into the gate material and shows very good agreement with experimental data on drift in OECTs. We show that the temporal current drift can be largely mitigated using a dual-gate OECT architecture and that dual-gate-based biosensors can increase the accuracy and sensitivity of immuno-biosensors compared to a standard single-gate design. Specific binding can be detected at a relatively low limit of detection, even in human serum.

## 1. Introduction

Organic electrochemical transistors (OECTs) are a reliable platform for biomolecule detection because of their low operation voltage and promising biosensing behavior. This type of device has drawn considerable attention in the biosensing domain [1,2,3,4]. The OECT device includes three terminals, which are source, drain, and gate terminals. The region between the source and drain terminals is called the channel region, which is usually covered by organic semiconductors (OSCs) or conductive polymers. A gate electrode is in contact with the channel region through the electrolyte, and OECTs are operated through the application of the gate voltage. The ions in the electrolyte are driven by this gate potential, which changes the doping state of the channel material and alters the ion–electron transport in the channel region [4,5,6,7]. N-type or p-type materials have been used in the OECT platform as channel materials, including optimized materials, such as poly(3-hexylthiophene-2,5-diyl) (P3HT) and poly((ethoxy)ethyl 2-(2-(2-methoxyethoxy) ethoxy)acetate)-naphthalene-1,4,5,8-tetracarboxylicdiimide-co-3,3′-bis(2-(2-(2-methoxyethoxy)ethoxy) ethoxy)-(bithiophene)) p(gNDI-g2T). Poly(3,4-ethylenedioxythiophene) doped with poly(styrene sulfonate) (PEDOT:PSS) is one of the most widely used materials in the OECT research field. This material has been used because it has high transconductance, which is beneficial for the behavior of the OECTs [7,8,9,10,11,12].

There are various categories of biomolecules that have been proven to be effectively detected through the OECT platform. Small biomolecules include sarcosine, urea, and glucose; relatively larger biomolecules including DNA or proteins; and even the incubation of cells can be detected through the OECT platform [13,14,15,16,17]. Based on the working principle of the OECTs, the amplified biosensing signals are related to high transconductance, which is related to the channel width, channel length, thickness of the channel material, and the type of channel material. Multiple avenues for optimizing OECT performance have been considered [18]. For example, by designing the structure of the channel material, via backbone or side-chain engineering, the electrical properties of the channel materials can be optimized [19]. Also, the behavior of ion-selective sensors can be improved by solid gel electrolytes [20]. Another option is to rearrange the alignment of the three terminals and create a new circuit design. The gate electrode can be deposited with source and drain electrodes on the same substrate, or the gate electrode can be placed on top of the channel region [16,21]. As for circuit design, it has been reported that a voltage-amplified circuit can be added to improve the behavior of OECTs. Integrated circuits, including multiple OECTs with data processed using associative learning, have also been reported to improve the accuracy of sensing signals [22,23,24]. Among single OECT-based biosensors, the reported lowest limited of detection (LOD) value is one molecule, which is achieved using a structure with a large-area gate electrode on top of a small-area channel region. By immobilizing the antibodies on a self-assembly monolayer on the gold gate electrode, an interaction between the antibody and antigen can be detected after just one single antigen molecule binds to the antibodies [16,25,26]. We have previously shown that this gate-over-channel structure can improve the behavior of biosensors, even without a gold electrode [27].

**Figure 1:**
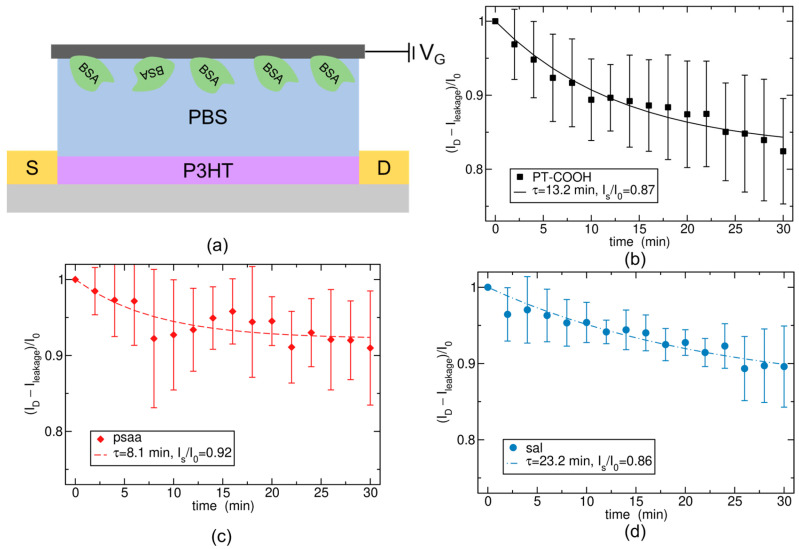
(**a**) Structure of the S-OECT platform. (**b**) Experimental and simulation data of PT-COOH-incorporated biosensors. (**c**) Experimental and simulation data of PSAA-incorporated biosensors. (**d**) Experimental and simulation data of SAL-incorporated biosensors. The data points show experimental data taken from ref. [27], and the curves show the theoretical model, Equation (11).

However, the drift phenomenon was consistently observed in the control experiments without any analyte present, which means that although there is no specific binding happening in the control experiment, we can still observe a temporal drift in the electrical signal [16,25,26,27]. The drift phenomenon can be largely canceled by a dual-gate sensing configuration (D-OECT), which we have reported for operation in a PBS buffer [27]. The D-OECT platform has two OECT devices connected in series; the gate voltage (V_G_) was applied from the bottom of the first device, and the drain voltage (V_DS_) was applied to the second device; the schematic image of the configuration is shown in Figure 1a. The transfer curves were measured from the second device. This design can prevent like-charged ion accumulation during measurement [27]. However, these previous results do not explore whether the D-OECT setup can still be effective in real biological fluids, such as blood serum. Moreover, there is a lack of experimental and theoretical analysis to demonstrate how the process of ion penetration and accumulation into the gate material causes the device drift.

Here, we quantitatively explain the origin of the drift phenomenon and demonstrate the practical significance of the D-OECT setup in real biological fluids. The drift phenomenon was first studied in 1X PBS solution, which is a much simpler system than real human serum. The biosensing platform was based on a single-gate sensing configuration (S-OECT), a configuration that has been previously reported to be subject to appreciable temporal drift in the output current [16,25,26,27]. We developed an analytical model using first-order kinetics, fit it to the new experimental data, and reanalyzed the raw data from the control experiment from our previous work [27]. The biomolecule used was human immunoglobulin G (IgG) since human IgG carries negative charges at the physiological pH value. The IgG antibodies were not immobilized on the gate electrode; only the bovine serum albumin (BSA) blocking layer was attached to the gate electrode to investigate the role of the ions and biomolecules on drift. Moreover, to understand the factors that influence ion penetration and accumulation, we investigated the influence of the BSA blocking layer and gate material thickness on the drift.

Furthermore, we investigated the performance of the double-gate (D-OECT) platform in real human fluid with poly [3-(3-carboxypropyl)thiophene-2,5-diyl] regioregular (PT-COOH) used as a bioreceptor layer with immobilized IgG antibodies. IgG in human serum was used as the target biomolecule to keep the species of the binding biomolecules consistent with our previous experiment in a phosphate-buffered saline (PBS) solution [27,28], thus enabling a quantitative comparison of the device sensing performance between PBS and human serum. Because human IgG is abundant in human serum, the biological fluid chosen for detection was human IgG-depleted human serum [29]. The reason for depleting the original human IgG in the human serum was to control the accuracy of the concentration of the human IgG in the system during the measurement.

## 2. Results and Discussion

### 2.1. Theoretical Modeling of the Drift Phenomenon in Single-Gate Functionalized Biosensors Based on BSA + Human IgG Control Experiment

Theoretical modeling was conducted based on the results of the BSA + human IgG control experiment, which was measured in 1X PBS using the S-OECT platform [23], which only has one functionalized gate and exhibits appreciable current drift. The structure of the S-OECT platform is indicated in Figure 1a. The first type of bioreceptor layer that we studied was PT-COOH, which is a p-type semiconducting polymer. Figure 1b illustrates the experimental and theoretical data for PT-COOH-incorporated biosensors. The experimental data are indicated by symbols with error bars, and the theoretical lines were obtained by fitting an exponentially decaying function (derivation provided below). At each subsequent experimental data point, the concentration of human IgG was ten times greater than for the previous point. However, in the absence of antibodies, the IgG was assumed to be irrelevant, and we hypothesized that the drift was only caused by small ions. Experiments without the presence of IgG will be discussed below. Figure 1c,d demonstrates the results using different bioreceptor layers, an insulating polymer poly (styrene–co–acrylic acid) (PSAA), and a self-assembly layer (SAL) [27]. The theoretical model was the same for all three types of bioreceptor layers; only the parameters changed.

The drift phenomenon was theoretically explained by the diffusion of the ions into the gate material. To simplify the problem, we only considered the dominant ions in a PBS solution, Na and Cl, and, at this point, disregarded low-molarity components such as K or PO_4_. We assumed that the ions could be absorbed into the bioreceptor layers, and at this point, the spatial distribution of ions in the material was neglected. The rate at which specific ions move from the solution to the bioreceptor layers is $k^{+}$, while the rate at which ions move from the bioreceptor layers to the solution is $k^{-}$. The relevant quantities are the ion concentration in the solution *c*_0_ and the ion concentration in the bioreceptor layers $c_{a}$. The change in ion concentration in the bioreceptor layers was thus determined by first-order kinetics,

(1) $$\frac{\partial c_{a}}{\partial t}=c_{0}k^{+}-c_{a}k^{-}.$$

We shall assume that $c_{0}$ was constant since the application used a high-ionic-strength solution (1X PBS solution or serum), and ion absorption was not expected to measurably affect $c_{0}$. The ratio of rate constants determines the equilibrium ion partition *K* between the solution and the gate material and is given by the electrochemical potential

(2) $$\frac{k^{+}}{k^{-}}=K=e^{-\frac{∆G+∆Ve_{0}z}{k_{B}T}}$$

where ∆G is the difference in the Gibbs free energy of an ion between the bioreceptor layer and the solution at no applied voltage, which is equal to the difference in the excess chemical potentials $∆G=-∆\mu^{ex}.$ ∆V is the difference in the electrostatic potential between the gate and the bulk solution, *e*_0_ is the unit charge, *z* is the ion valency (an integer), *k*_B_ is the Boltzmann constant, and *T* is the absolute temperature. The base rate, *k*_0_ = $k^{-}$(∆G = 0, ∆V = 0), was determined by the diffusion constant *D* of ions in the bioreceptor layer but also the width of the layer *d* within which ions could be incorporated into the material. We can estimate *k*_0_ ~ *D*/*d*^2^, which measures the typical time for the ions to diffuse into or out of the layer of width *d*. We assumed that the absorbed concentration was small compared to the solvent ($c_{a}<c_{0}$). Note that this formulation does not consider the spatial distribution of ions, which could be obtained using the Nernst–Planck equation and a finite element solver. Contrary to the Nernst–Planck equation, the simpler kinetic formulation considered here permits an analytical prediction at low densities of absorbed ions.

Due to ion absorption, the material solution interface could be considered to have a capacitance per unit area *C*_A_~ε/d, with ε being the dielectric constant in the bioreceptor layer. The electrostatic potential is not a constant but depends on the concentration of adsorbed ions $c_{a}$,

(3) $$∆V=∆V_{0}+\frac{c_{a}ze_{0}d}{C_{A}},$$

where $∆V_{0}$ is the applied voltage between the gate electrode and the bulk solution, and $c_{a}ze_{0}d$ is the absorbed charge per unit area. We assume that for $∆V_{0}>0$, anions are absorbed (z=−1 for monovalent anions), whereas for $∆V_{0}<0$, cations are absorbed (z=1, assuming cations are monovalent), such that the product $∆V_{0}e_{0}z$ is always negative, $∆V_{0}e_{0}z<0$.

The differential equation that describes the evolution of absorbed charge $c_{a}$ (and electrostatic potential ∆V) is thus obtained by inserting Equations (2) and (3) into (1),

(4) $$\frac{{\partial c}_{a}}{\partial t}=k_{0}\left(c_{0}e^{-\frac{∆G+\left(\frac{c_{a}d}{C_{A}}\right){\left(e_{0}z\right)}^{2}+{∆V}_{0}e_{0}z}{k_{B}T}}-c_{a}\right).$$

This equation is not analytically solvable. However, for small, absorbed ion densities where $c_{a}d{\left(e_{0}z\right)}^{2}<C_{A}k_{B}T$, the exponential can be expanded to the first order, yielding

(5) $$\frac{{\partial c}_{a}}{\partial t}=k_{0}\left(c_{0}e^{-\frac{∆G+∆V_{0}e_{0}z}{k_{B}T}}\left[1-\frac{c_{a}d{\left(e_{0}z\right)}^{2}}{C_{A}k_{B}T}\right]-c_{a}\right),$$

which, for a constant applied gate voltage $∆V_{0}$, can be analytically integrated. For an initial condition with no ions in the bioreceptor layer, $c_{a}\left(t=0\right)=0$, the evolution of ion density follows an exponential approach to a steady-state value $c_{s}$,

(6) $$c_{a}\left(t\right)=c_{s}\left(1-e^{-\frac{t}{\tau }}\right),$$

where the steady-state value is

(7) $$c_{s}={c_{0}\left(e^{\frac{∆G+∆V_{0}e_{0}z}{k_{B}T}}+\frac{c_{0}d{\left(e_{0}z\right)}^{2}}{C_{A}k_{B}T}\right)}^{-1}$$

and the timescale is

(8) $$\tau^{-1}=k_{0}\left(1+\frac{c_{0}d{\left(e_{0}z\right)}^{2}}{C_{A}k_{B}T}e^{-\frac{∆G+∆V_{0}e_{0}z}{k_{B}T}}\right).$$

Since we know the evolution of $c_{a}\left(t\right)\;$ we can predict the evolution of the surface electrostatic potential via Equation (3),

(9) $$∆V\left(t\right)=∆V_{0}+\frac{c_{a}\left(t\right)ze_{0}d}{C_{A\;}}.$$

Finally, we consider the fact that the changes in the surface potential due to ions are small, such that the transfer curves of the OECT can be approximated as linear,

(10) $$I_{D}\left(t\right)-I_{0}=B\left(∆V\left(t\right)-∆V_{0}\right),$$

where *I*_D_(*t*) is the drain current, *I*_0_ is the initial drain-source current at gate voltage ∆V_0_, and *B* is the transconductance (actually, transconductance is the absolute value of *B*, since *B* is negative if $∆V_{0}$ is negative). Thus, the predicted source-drain current drift over time due to ion absorption into the gate materials is

(11) $$\frac{I_{D}\left(t\right)}{I_{0}}=1+\frac{Bdze_{0}}{{I_{0}C}_{A}}c_{a}\left(t\right)=\frac{I_{S}}{I_{0}}+e^{-\frac{t}{\tau }}\left(1-\frac{I_{S}}{I_{0}}\right),\;$$

where τ is given by Equation (8), and the steady-state current is

(12) $$\frac{I_{S}}{I_{0}}=1+\frac{Bdze_{0}c_{s}}{I_{0}C_{A}}.\;$$

Note that the second term is negative (since $∆V_{0}z<0$ and Bz<0), and $c_{s}$ is given by Equation (7).

Since we do not know the values of quantities in Equations (8), (9) and (12), we treat *I*_s_ and τ as fitting parameters. We find that the experimental data for three different gate materials can be well explained by Equation (11), which is indicated in Figure 1. The three materials exhibit different values for *I*_s_ and τ. Differences in *I*_s_ can arise due to different ion absorption-free energy ∆*G*, depending on different porosities of the materials or chemical affinity for the ions, due to different capacitance *C*_A_, which depends on the depth *d* to which the ions are penetrating, or due to a different transfer function coefficient, *B*, as stated in Equation (12). Likewise, differences in the timescale τ can arise due to different ion diffusivities in the materials (via *k*_0_) or due to a different penetration depth, *d*, as stated in Equation (8).

Importantly, the experimentally obtained drifts (Figure 1) are well explained by the exponential approach to a steady state (Equation (12)). This suggests that output from a single-gate device could be corrected for this exponential drift, increasing the sensitivity of the single-gate device. Moreover, this theory also suggests that a double-series gate configuration would yield two opposing exponential drifts. If the parameters *I*_s_ and τ for both cations and anions responsible for the drift are very similar, the two drifts will largely cancel out. Thus, a successful double-series gate application likely requires a gate material that has approximately equal affinities for both cations and anions.

In the theoretical treatment above, we only considered the major components of PBS, Na^+^, and Cl^−^ ions. PBS and serum contain other multivalent ions such as phosphate. Drift in the presence of adsorption of ions of multiple types will be investigated in future work. Our model assumes that the ion penetration into the gate only changes the electrostatic potential, while the dielectric constant remains constant. However, dielectric constant may also change, but that change is likely symmetric; the same applies for cations and anions, and thus, would not be canceled by the dual-gate configuration. Since we observed that the dual-gate configuration did largely cancel the drift in PBS [27], we concluded that the dielectric effects were not dominant.

### 2.2. Experimental Study of the Drift Phenomenon in Single-Gate Functionalized Biosensors

**Figure 2:**
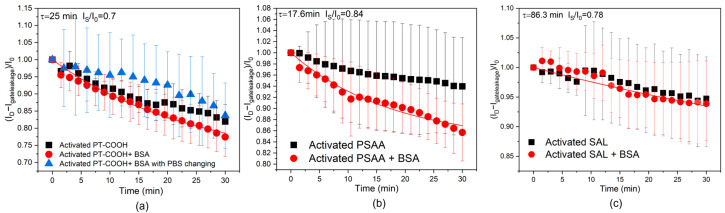
Current drift for various OECT configurations. (**a**) PT-COOH-incorporated biosensors with PBS changing steps, activated PT-COOH-incorporated biosensors, and activated PT-COOH + BSA-incorporated biosensors. (**b**) Activated PSAA-incorporated biosensors and activated PSAA + BSA-incorporated biosensors. (**c**) Activated SAL-incorporated biosensors and activated SAL + BSA-incorporated biosensors. The solid curves show the theoretical fit with parameters τ and *I*_s_ (Equation (11)).

Except for modeling the possible ion diffusion process that happened in the control experiments shown in Figure 1, where IgG was present, whether human IgG can interfere with the ion diffusion or if the blocking layer (BSA layer) can influence ion accumulation needs to be considered. In this case, another set of control experiments without the addition of human IgG was conducted. The measurement setup was still based on the S-OECT platform, but the electrolyte was pure 1X PBS without human IgG, and the measurement was conducted with the same timescale as the BSA+ IgG control experiment discussed above. For PT-COOH-incorporated biosensors, the influence of the solution-changing step was also investigated. During the measurement, the electrical signal output might have also been influenced by the process of adding different concentrations of analyte solutions; therefore, the 1X PBS solution was also repeatedly removed and added to the electrolyte to evaluate the influence of this process. The results for all these experiments are indicated in Figure 2.

Figure 2a indicates the results with and without the PBS changing step. The PBS changing step mimics the measurement procedure of the control experiment, and without the changing step, we investigated the same PBS solution still being left in the device. It can be deduced that continuously removing and adding the PBS solution process did not significantly affect the output current; the error bars of the two sets of results largely overlapped. Comparing the current ratio change from Figure 1b and Figure 2a, the control experiment (with human IgG in PBS) has an average minimum drift of 0.83, while the current ratio drift of activated PT-COOH + BSA in PBS is 0.77, and the current ratio drift of activated PT-COOH in PBS is 0.82. These results indicate that the presence of human IgG is not the key factor that influences the scale of the drift. This matches with the conclusion from the modeling section, indicating that the drift phenomenon is dominated by the diffusion and accumulation of the ions from the PBS solution.

Since BSA carries negative charges in a PBS solution, the electrostatic forces between BSA and the ions may be favorable for ion penetration [30]. While BSA does not appreciably affect the drift for PT-COOH, the function of BSA is more obvious in PSAA-OECTs. Figure 2b shows that for activated PSAA-OECTs, the current ratio drift is 0.94, while for BSA+ activated PSAA, the current ratio drift is 0.86. After the attachment of the BSA blocking layer, the average current ratio shows a relatively obvious decrease. Since PSAA is an insulating polymer, even after the activation step, the sites available for ion accumulation may still be limited, but BSA may increase the number of sites that ions can penetrate. The ions may be attracted to BSA and be stabilized close to the activated PSAA on the gate electrode. Also, the fitting based on Equation (11) still works for these set of experiments. For SAL-OECTs (shown in Figure 2c), the current drift with and without the blocking layer does not shows any differences and is also close to the scale of the current ratio decrease in the control experiment, which is between 0.9–0.95. This may be because the SAL has a more regulated and lower density of carboxylic acid groups; therefore, the ability of BSA to increase the pathways of the ions is also limited.

Comparing the three types of bioreceptor layers, it can be seen that the PBS changing process and the existence of human IgG are not the crucial factors that cause the drift of the current. Instead, we conclude that the continued penetration and accumulation of the ions in the bioreceptor layer is the main cause of the drift phenomenon. As we will now show through an additional experiment, the drift is more pronounced when using thicker gate materials, and we explain that thinner gate materials reduce the steady-state magnitude of the drift due to smaller interfacial capacitance (Equation (12)).

**Figure 3:**
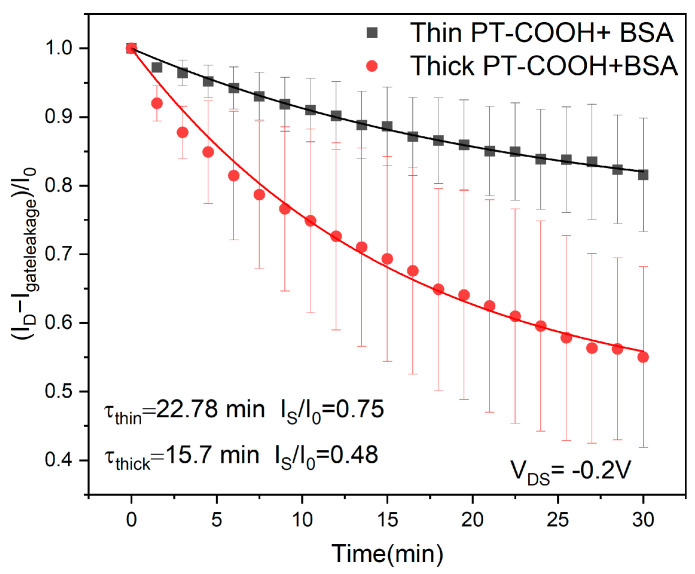
Current drift for PT-COOH-incorporated biosensors at different PT-COOH thicknesses: 55 nm (thin) and 112 nm (thick). The curves show the theoretical fit with parameters τ and *I*_s_ (Equation (11)).

To investigate the effect of the thickness of the gate material, we prepared two different PT-COOH gate materials with thicknesses of 55 nm and 112 nm. The change in the PT-COOH film thickness was controlled by the spin-coating speed, and the concentration of the PT-COOH solution remained the same. Figure 3 shows the drift behavior of BSA-attached PT-COOH films with different thicknesses. It can be deduced that the larger thickness of the bioreceptor film leads to larger drift. Specifically, the observed change in the steady-state drift is proportional to thickness, $1-I_{S}/I_{0}\propto d$, as predicted by Equation (12) at constant capacitance.

The observed drift timescales of the two layers (22 min vs. 15 min for the thinner and thicker layers, respectively) are of the same order of magnitude. The precision of this timescale comparison is limited by increasing uncertainty at longer drift times.

### 2.3. Dual-Gate-Functionalized Biosensor Behavior in a Real Biological Fluid

**Figure 4:**
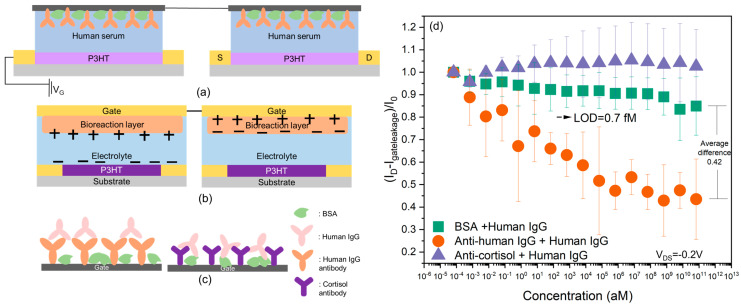
(**a**) Structure of the D-OECT platform. (**b**) Schematic image of opposite polarity created by D-OECT platform; a schematic circuit diagram is shown in Figure S5. (**c**) Schematic image of interactions on the gate electrode (IgG binds to the correct antibody but not to the incorrect antibody). (**d**) Current change in the main (orange circles) and control experiments (purple triangles, green squares) based on the D-OECT platform measured in IgG-depleted human serum. I_gateleakage_ is the gate leakage current, and I_0_ is the drain-source current at minimum gate voltage measured in human IgG-depleted human serum without an analyte (IgG).

Lastly, we investigated the drift in a dual-gate (D-OECT) platform. The D-OECT configuration consists of two OECT devices connected in series with opposite polarity (Figure 4a,b). Photographs and circuit schematics of the device are shown in Figures S4 and S5. Both gates were functionalized with bioreceptors—in this case, human IgG antibodies [23]. Figure 4c illustrates the antigen–antibody interaction that happens on the gate electrode. Figure 4d shows the results measured in IgG-depleted human serum based on the D-OECT platform with PT-COOH as the bioreceptor layer. I_D_ represents the drain-source current at the minimum gate voltage (maximum in absolute value) measured at different concentrations of human IgG. For the main experiment, there was a specific binding between human IgG antibody and human IgG, while for the control experiment, cortisol antibody was used, which was not to have a specific binding to IgG.

The D-OECT setup was designed to largely eliminate the drift phenomenon. Since two gates were connected in series, the applied voltage created opposite polarity on the two gate interfaces. Therefore, oppositely charged ions accumulated at the two interfaces or bioreaction layers during the measurement. The drifts at the two interfaces could significantly cancel out, which is why this D-OECT configuration could largely eliminate the drift from in 1X PBS [27]. It can be seen from Figure 1 that in the control experiments (IgG interacting with a mismatched antibody or no antibody), a small drift was still present in human serum. For the BSA+ IgG control experiment, the decreasing tendency of the current ratio was relatively stable at the low-concentration region but became more obvious when the concentration reached 100 ng/mL. The maximum average change in the current ratio reached values of 0.84. Compared with the results measured in 1X PBS [27], the drift was slightly larger. The larger drift may be a consequence of the more complex composition of the human serum. The human serum not only contains ions but also other small or large biomolecules including hormones and metabolites [31]. Although the charges carried by the ions can be largely neutralized through the opposite current flow direction, the charges carried by the biomolecules may not be fully canceled by the system. The size of the biomolecules and the polarity of the biomolecules can influence the accumulation of the biomolecules on the gates, which may not be symmetrical on both gates [32,33]. In the device schematic of Figure 4, the following consolidation would be possible. The extra “source” electrode on the left (blank) slide could be omitted, and the two “gates” could be functionalized polymer surfaces on the opposite sides of a conductive substrate. This would be electronically equivalent. However, the two oppositely aligned gate interfaces and the opposite polarity of the two P3HT films are essential for the most complete drift cancellation.

The cortisol antibody + human IgG control experiment data appear essentially stable and somewhat different from the BSA control, but not statistically distinguishable. This may be due to the intrinsic cortisol and polar biomolecules in the human serum [31,34]. Cortisol (which might have been in the serum) can have an interaction with the cortisol antibody, and polar biomolecules can also accumulate close to the gate electrode, which can change the surface potential of the gate electrode. The rearrangement of the charges can cause a small change in the current ratio [35]. Considering the main experiment, the level-of-detection (LOD) value was 0.7 fM, which was calculated using a method indicating that the difference between the average current ratio of the control experiment and the main experiment should be at least three standard deviations [16,36]. While this is already a clinically useful sensitivity for some applications, the LOD value that we obtained in human serum was considerably larger than the value we previously measured in 1X PBS, which was 0.07 aM using the D-OECT and 7 aM using the S-OECT [27]. More specifically, we carried out *t*-tests between the control and the main experiments (in Figure 4), and the result shows an obvious difference between the control and the main experiment after the concentration reached 67 aM, *p*-value < 0.05, which means that our system has the ability to differentiate specific binding from the noise. This is consistent with the hypothesis that the biomolecules in the system can complicate the behavior of the D-OECT-based biosensor. Thus, further research into the mechanisms of serum components contributing to the drift and designs of materials to minimize this will be the goal of future work.

## 3. Materials and Methods

### 3.1. Bioreceptor Layer Fabrication and Biomolecule Immobilization

Details of the fabrication and immobilization process can be found in the reference to our prior work [23]. PT-COOH and PSAA were heated and dissolved in dimethylformamide (DMF), and spin-coated with a concentration of 15 mg/mL. SAL was fabricated by immersing the indium-doped tin oxide (ITO)/poly(ethylene terephthalate) (PET) substrate in a 70 mg/mL 1,10-decanedicarboxylic acid (DDA) solution. DDA was dissolved in ethanol. To immobilize the human IgG antibody, the COOH groups of bioreceptor layers were activated through (N-(3-dimethylaminopropyl)-N′-ethylcarbodiimide hydrochloride (EDC) and N-hydroxysulfo- succinimide sodium salt(NHS)(EDC/NHS) chemistry; then, the human IgG antibody solution was left while still on the activated bioreceptor layers [27]. For the comparison of the two PT-COOH thicknesses, the spinning time was 320 s, which is the same spinning time as for our main experiment The spinning speed was 3000 rpm for the 55 nm layer (with ±11 nm) and 800 rpm for the 112 nm (width ±20 nm) layer. After spin coating, the films were heated in a vacuum oven at 60 °C for at least four hours to remove the residual DMF.

### 3.2. OECT Measurements

OECT devices have a channel length of 1 mm and a channel width of 20 μm. The total area of the channel region is 1.25 mm^2^, and the area of the functionalized gate is 1 cm^2^. The transfer curves were measured at V_DS_ = −0.2 V, and V_G_ was swept from 0.1 V to –0.7 V. In the dual-gate system, the effective voltage from the original P3HT-coated substrate to the first gate and from the second gate to the P3HT channel would be about 0.05 to 0.35 V during the sweep, about half the nominal applied Vg to the total circuit, since the materials in the electronic pathways at each gate are the same. For the experiment conducted in human serum based on D-OECT configuration, human IgG was dissolved and diluted in human serum, the electrolyte well of the first device was filled with human IgG-depleted human serum, and the electrolyte well of the second device was filled with human serum and human IgG. The bioreceptor layers were attached to the gate electrode, and the channel region of the two devices was covered by P3HT. A stabilization process was conducted before changing the concentration of the analyte [27]. The dilution process was conducted from the highest concentration to the lowest. We initially dissolved the antigen (IgG, obtained in solid form) into the PBS solution or the commercially available human serum at a concentration of 10 mg/mL and then diluted that solution sequentially using a metered pipette to obtain solutions at all the other concentrations. This process was identical for all experiments that used IgG. The transfer curves were measured with V_DS_ = −0.2 V, with V_G_ being swept from 0.1 V to −0.7 V.

The drift study experiments were conducted based on the S-OECT platform, and the electrolyte well was filled with 1X PBS. For S-OECT, the biomolecules were functionalized on the gate, and the channel region of the OECT devices was covered by P3HT [23]. The first type of experiment was conducted on activated-bioreceptor-layer-incorporated OECTs. All three types of bioreceptor layers were activated through EDC/NHS chemistry, and then the PBS solution was left while still in the electrolyte well for the repeated measurement of transfer curves. The other type of experiment was conducted on BSA + activated-bioreceptor-layer-incorporated OECTs. For the PBS changing experiments on PT-COOH-OECTs, the number of transfer curves measured was the same as the number of transfer curves measured in the control experiment. For the measurements without the PBS changing step, the same PBS solution was also left still in the electrolyte, and the transfer curves were repeatedly measured at the same timescale as the control experiments. For the drift study of all three types of bioreceptor layers, the transfer curves were measured without the PBS changing step.

## 4. Conclusions

In summary, we investigated the physical origin of the drift phenomenon, which is common in OECT-based biosensors and was observed in our S-OECTs. By using theoretical modeling and experimental methods, we deduced that the dominant factor that causes the current drift is the accumulation and penetration of ions into the bioreceptor layer. We showed that thinner gate materials reduce the steady-state magnitude of the drift due to smaller interfacial capacitance. The diffusion process of the ions from the solution to the bioreceptor layer follows first-order kinetics. If the transport properties of cations and anions are the same, the double-gate setup should lead to the cancelation of the drift. However, this theory is based on the buffer solution; if the biosensing environment becomes more complex, for instance, including various types of multivalent ions and biomolecules that may add additional drift, the cancelation will not be perfect.

In addition, we demonstrated the biosensing capabilities of the D-OECT platform in human serum, which broadened the range of applications for the D-OECT platform. This suggests that the D-OECT platform can be applied in human serum. Although the behavior of the D-OECT-based immuno-biosensors can be further optimized, the current device data already show that this type of biosensor can be used to detect the interaction between biomolecules in real biological fluids.

A previous publication [8] considered the stability of the semiconducting polymer in OECTs in aqueous media. Two results from that manuscript support our approach. First, the drift reported in the reference appeared to be chemical in origin, not caused by an electrochemical gating operation, and was in the same direction, despite the differences in salt content and immersion times. Second, the drift was associated with threshold voltage shifting rather than mobility, which is consistent with an interfacial potential drift that we worked to understand and compensate for in the present manuscript. On the other hand, there are several important differences between this reference and our work. First, the focus of our manuscript is on the activity in functionalized polymer carboxylates used as gating materials, which is much more chemically active than P3HT and would dominate the various drift mechanisms. Second, some observations reported in the reference may relate to the SiO_2_ dielectric layer, while in our system, gold electrodes were coated on the glass substrate. Third, we were concerned with buffer analyte solutions and serum, rather than pure water. Fourth, our approach was to develop a means of electronically compensating for the drift.

Organic mixed ionic–electronic conductors have drawn increasing attention due to their high ionic sensitivity [9,10,11]. OECTs can be operated in depletion mode, which stands for channel current decreasing with larger gate potential; OECTs can also be operated in accumulation mode, which stands for channel current increasing with larger gate potential [7,11,12,13]. Materials applicable to OECTs are increasingly applied to biosensors [37,38,39,40,41].

The future direction of using the D-OECT platform should not only exclude the influence of the ions but also other biomolecules in human serum. The results indicate that the LOD value in human serum is relatively large, which means that the specific binding and the binding detecting process may be influenced by the internal biomolecules in serum. Another possibility is that multivalent ions cause part of the drift. Since serum typically contains a higher concentration of multivalent cations, such as Ca^2+^ and Mg^2+^, compared to multivalent anions, the drift due to multivalent ions may not be fully canceled by the D-OECT configuration. The route to further minimize drift due to the combined action of multiple ions and charged biomolecules in a D-OECT setting will be studied in a future research work. In this way, the influence of biomolecules or ions can be further understood, and the behavior of the D-OECT-based biosensors can be further improved, for example, by using two different gate materials or materials with different thicknesses that are optimized for drift cancelation over the relevant detection range.

## Data Availability

Original data will be posted to the Johns Hopkins Research Data Repository, https://archive.data.jhu.edu/dataverse/root?q= (accessed on 23 February 2024).

## Supplementary Materials

The following supporting information can be downloaded at https://www.mdpi.com/article/10.3390/molecules29071459/s1, Figure S1: (a) Transfer curves of drift study with activated PT-COOH and BSA incorporated S-OECTs. (b) Transfer curves of drift study with activated PSAA and BSA incorporated S-OECTs. (c) Transfer curves of drift study with activated SAL and BSA incorporated S-OECTs. Figure S2: Biosensing results based on the D-OECT platform with PT-COOH as the bioreceptor layer. Figure S3: An additional result from [24]. We used a self-assembly layer (SAL) as the bioreceptor layer instead of polymer layers. The left-side figure represents the results measured on single gate biosensors and the right-side figure represents the results measured on dual gate biosensors. It can be seen that the D-OECT can decrease the drift even with SAL, not only with the polymer layers. Figure S4: Photographs of original channel substrate and dual gate device. Figure S5: Schematic circuit diagram corresponding to the dual gate device in Figure 4. Figure S6: Transfer curves measured based on D-OECT platform in human serum with human IgG concentration change.
