# Supplementary material for: A Study of the Drift Phenomena of Gate-Functionalized Biosensors and Dual-Gate-Functionalized Biosensors in Human Serum
Source: Molecules. 2024 Mar 25;29(7):1459. doi: 10.3390/molecules29071459 (PMC11013244; doi:10.3390/molecules29071459)
Supplement: Supplementary file 1 [file molecules-29-01459-s001.zip › molecules-2908600-supplementary.pdf]

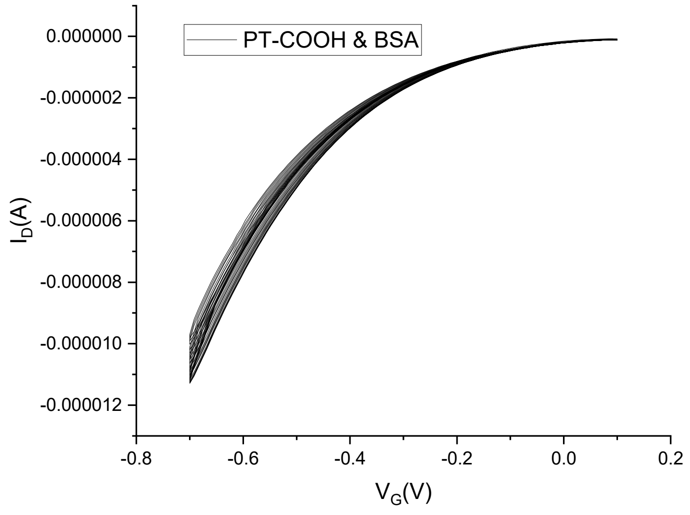
PT-COOH & BSA
0.000000
-0.000002
-0.000004
-0.000006
-0.000008
-0.000010
-0.000012
I_D(A)
-0.8
-0.6
-0.4
-0.2
0.0
0.2
V_G(V)

(a)

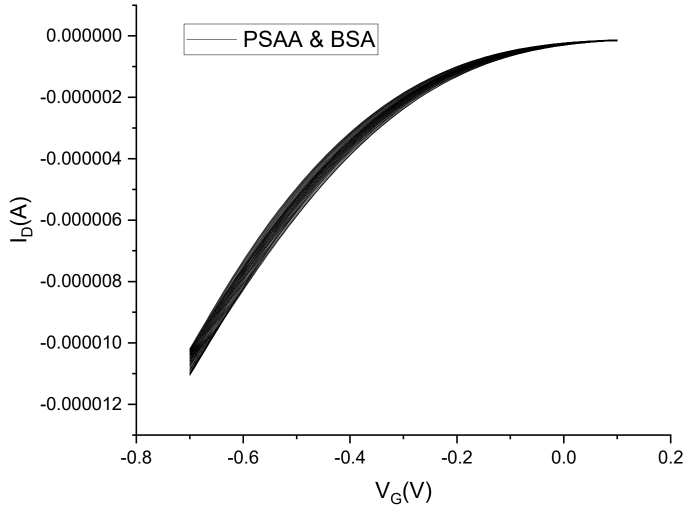
PSAA & BSA
0.000000
-0.000002
-0.000004
-0.000006
-0.000008
-0.000010
-0.000012
I_D(A)
-0.8
-0.6
-0.4
-0.2
0.0
0.2
V_G(V)

(b)

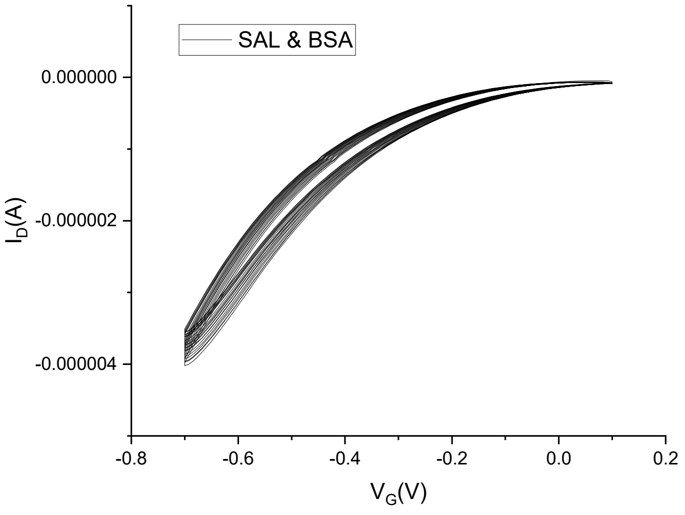

(c)

Figure S1. (a) Transfer curves of drift study with activated PT-COOH and BSA incorporated S-OECTs. (b) Transfer curves of drift study with activated PSAA and BSA incorporated S-OECTs. (c) Transfer curves of drift study with activated SAL and BSA incorporated S-OECTs.

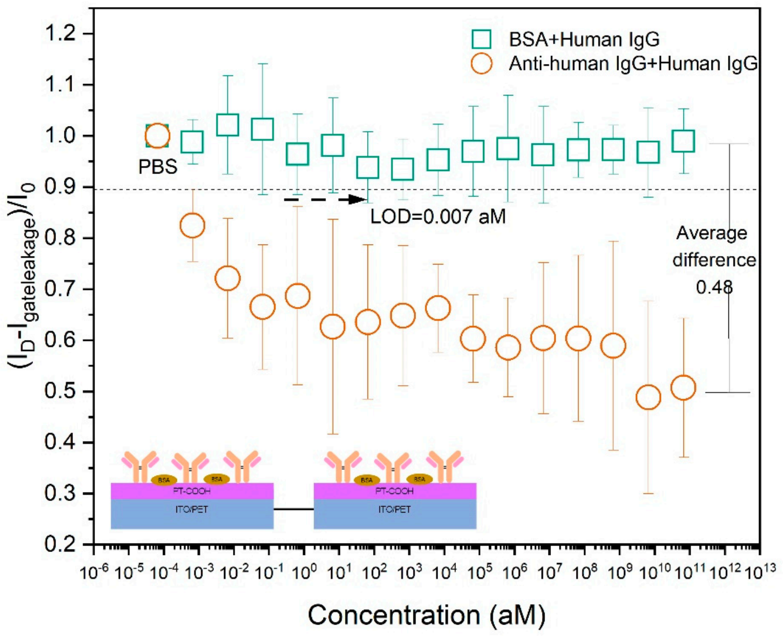

Figure S2. Biosensing results based on the D-OECT platform with PT-COOH as the bioreceptor layer.[24]

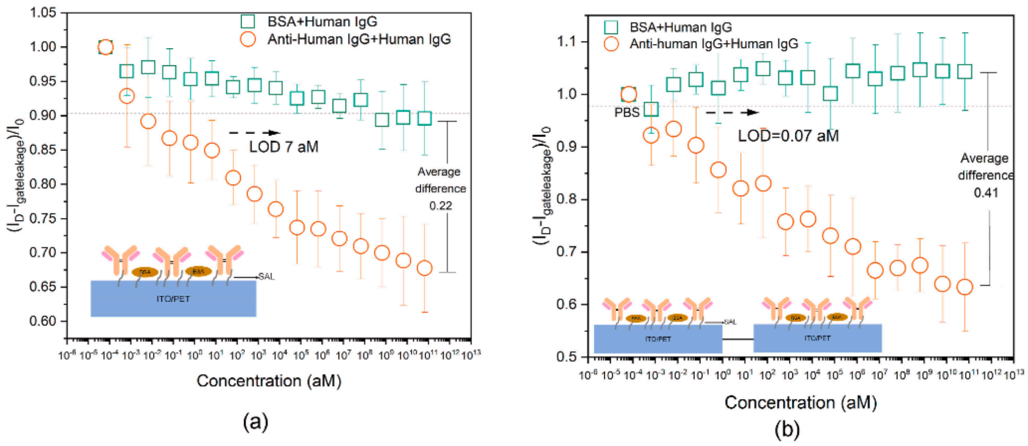

Figure S3. An additional result from reference 24. We used a self-assembly layer (SAL) as the bioreceptor layer instead of polymer layers. The left-side figure represents the results measured on single gate biosensors and the right-side figure represents the results measured on dual gate biosensors. It can be seen that the D-OECT can decrease the drift even with SAL, not only with the polymer layers.

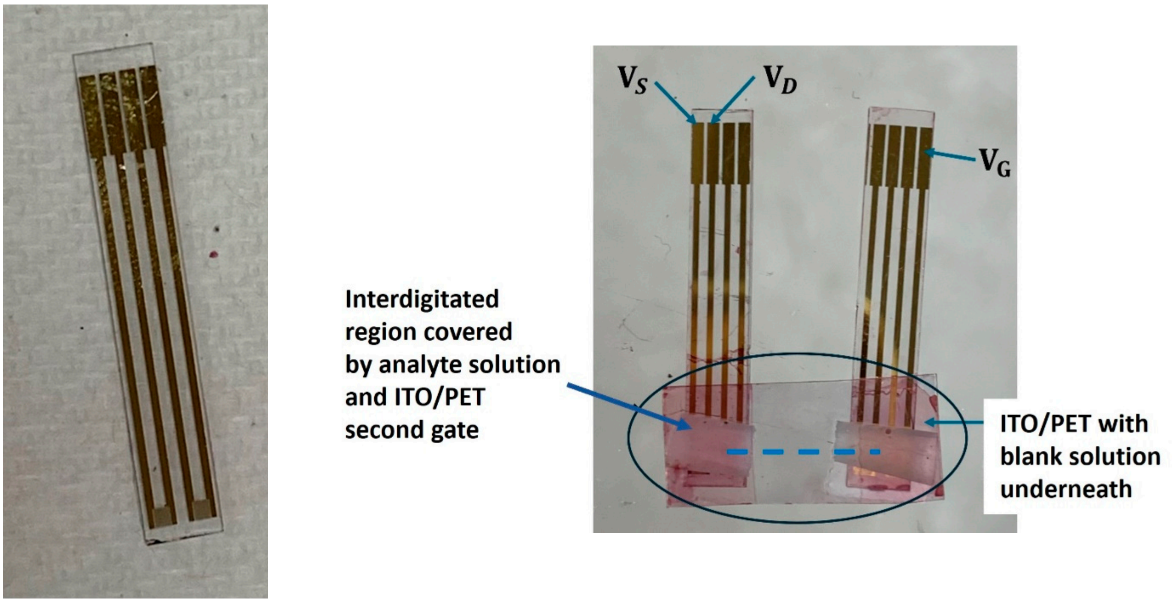

Figure S4. Photographs of original channel substrate (left) and dual gate device (right). The interdigitated electrodes contacting the channel are at the very bottom of the gold lines. Two channels are on each substrate, and one channel per substrate is used at a time. The gate voltage is applied to one gold pad (upper right in the right photograph) and is transmitted through a blank solution, an indium tin oxide (ITO) layer on poly(ethylene terephthalate) (PET), a wire, a second piece of ITO/PET, and the analyte solution to reach the channel on the left. Interdigitated electrodes (NanoSPR) had 150nm gold patterned on 3 cm-long silica substrates; total width 20 mm; length 10 μm.

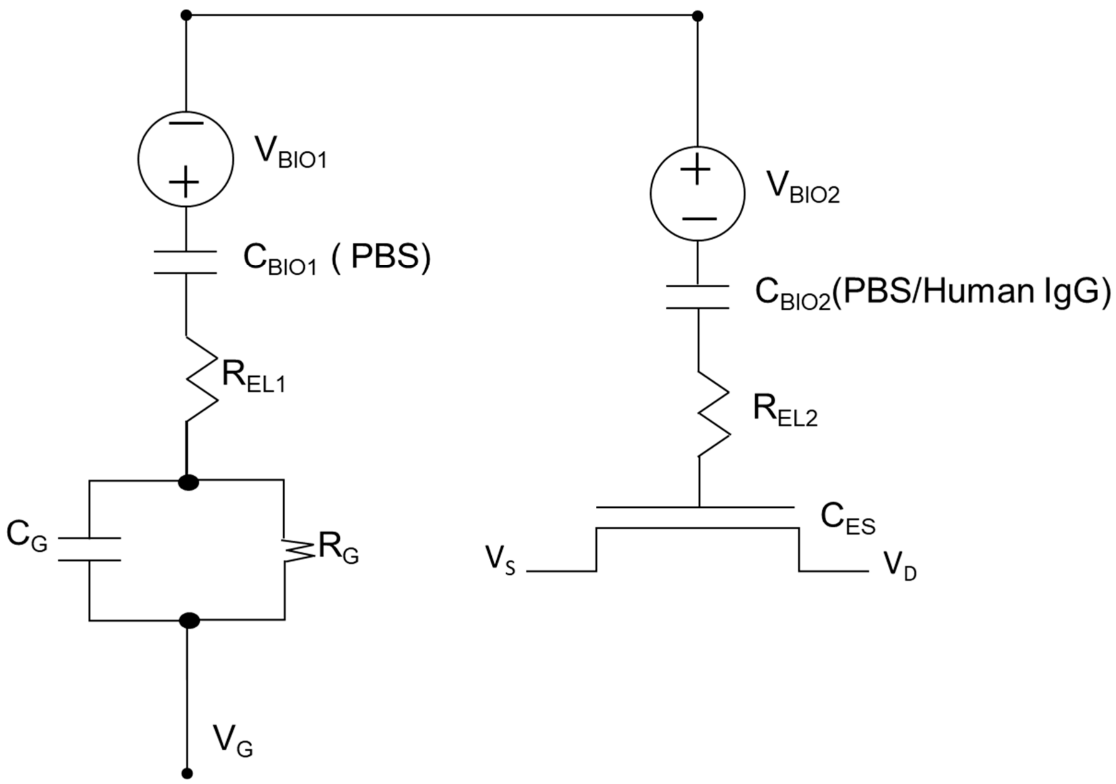

Figure S5. Schematic circuit diagram corresponding to the dual gate device in Figure 4. For Figures S2, S3, and S5, copyright Elsevier. Used with permission.

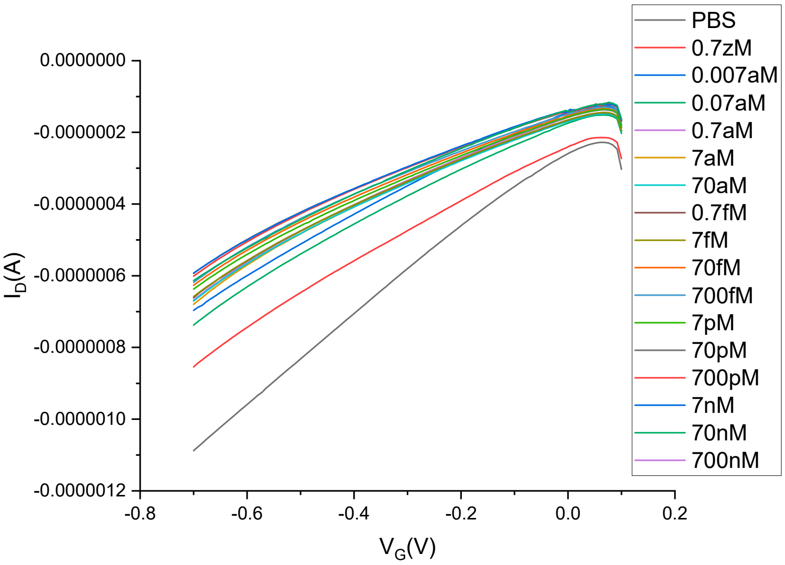

Figure S6. Transfer curves measured based on D-OECT platform in human serum with human IgG concentration change.
